# Institutional Questions

**Published:** 1900-02-24

**Authors:** 


### INSTITUTIONAL QUESTIONS.

### Wards for Consumption.

"ARCHITECT" asks: Kindly advise me on the following points: 1. It is desired, in the case of a new general hospital about to be erected, to provide a certain number of beds for consumption cases. Would it be considered altogether a mistake to arrange a ward for such cases under the same roof as the other wards; say, on the top floor? 2. Or would the provision of a separate pavilion in the grounds remove any possible objection which might be raised by those who hold to the complete isolation of these cases?

*** 1. There are many people who would certainly condemn the plan of placing a ward for consumptives on the top floor of a general hospital, the whole question of the treatment of such cases being the subject of much controversy at the present time. We should say, however, that looking at the improved methods of hospital construction obtaining now, that given solid walls, and with the extreme precautions now taken to prevent aerial inter-communication between the floors, there can be no real reason why the plan you propose should not be followed with perfect safety to all concerned. At the same time, to answer your second point (2), all criticism would be avoided if it were possible to provide the required beds in a separate pavilion. This is undoubtedly the ideal method, but it is of course a more expensive proceeding. The question will no doubt resolve itself into one of £ s. d.

### Disinfection by Burning.

"J. B." writes: Can you tell me anything of a process used, I understand, on the Continent, of disinfecting hospital wards or pavilions by "burning out" the interiors. Does this mean the entire destruction of the building, or in what way can the process be carried out without injuring the structure?

*** Your question would seem to refer to two systems. The plan advocated by Sir James Simpson in his book "Hospitalism" was the entire destruction of hospital buildings after, say, ten years. This meant, of course, the erection of buildings only intended to be of a temporary character. Another plan of disinfection by fire is that urged by the late Sir Benjamin Richardson, who in his "Hygeia," recommended that the walls of hospital wards should be of metal, and that these should be purified by the application of fire in the same way that paint is now treated, but in less drastic fashion, by the ordinary workman when redecorating. The whole question of the desirability of destroying hospital buildings periodically was thoroughly gone into some years ago, under the direction of the Local Government Board, with the result that the plan of erecting temporary buildings, and burning them after a certain number of years, was definitely condemned by that authority, and the granting of loans for the erection of temporary isolation hospitals is not allowed. We have no information with reference to any plan specially carried out on the Continent in this connection.

### THE DEACONESSES HOME, BAARN.

MONS. L. C. BOISSVAN is sole architect of the above institution, and he did not plan it in partnership with another firm of architects, as was stated by mistake in our notice.

